# Recent Development of Fluorescent Nanodiamonds for Optical Biosensing and Disease Diagnosis

**DOI:** 10.3390/bios12121181

**Published:** 2022-12-19

**Authors:** Shahzad Ahmad Qureshi, Wesley Wei-Wen Hsiao, Lal Hussain, Haroon Aman, Trong-Nghia Le, Muhammad Rafique

**Affiliations:** 1 Department of Computer and Information Sciences, Pakistan Institute of Engineering and Applied Sciences (PIEAS), Islamabad 45650, Pakistan; 2 Department of Chemical Engineering, National Taiwan University of Science and Technology, Taipei 106, Taiwan; 3 Department of Computer Science and Information Technology, King Abdullah Campus Chatter Kalas, University of Azad Jammu and Kashmir, Muzaffarabad 13100, Pakistan; 4 Department of Computer Science and Information Technology, Neelum Campus, University of Azad Jammu and Kashmir, Athmuqam 13230, Pakistan; 5 School of Mathematics and Physics, The University of Queensland, St Lucia, QLD 4072, Australia; 6 National Institute of Lasers and Optronics College, PIEAS, Islamabad 45650, Pakistan; 7 Institute of Atomic and Molecular Sciences, Academia Sinica, Taipei 106, Taiwan; 8 Department of Physics, King Abdullah Campus Chatter Kalas, University of Azad Jammu and Kashmir, Muzaffarabad 13100, Pakistan

**Keywords:** fluorescent nanodiamonds, nanosensors, intracellular thermometry, nanoscale magnetometry, ultrasensitive biosensing, machine learning, biomarker detection

## Abstract

The ability to precisely monitor the intracellular temperature directly contributes to the essential understanding of biological metabolism, intracellular signaling, thermogenesis, and respiration. The intracellular heat generation and its measurement can also assist in the prediction of the pathogenesis of chronic diseases. However, intracellular thermometry without altering the biochemical reactions and cellular membrane damage is challenging, requiring appropriately biocompatible, nontoxic, and efficient biosensors. Bright, photostable, and functionalized fluorescent nanodiamonds (FNDs) have emerged as excellent probes for intracellular thermometry and magnetometry with the spatial resolution on a nanometer scale. The temperature and magnetic field-dependent luminescence of naturally occurring defects in diamonds are key to high-sensitivity biosensing applications. Alterations in the surface chemistry of FNDs and conjugation with polymer, metallic, and magnetic nanoparticles have opened vast possibilities for drug delivery, diagnosis, nanomedicine, and magnetic hyperthermia. This study covers some recently reported research focusing on intracellular thermometry, magnetic sensing, and emerging applications of artificial intelligence (AI) in biomedical imaging. We extend the application of FNDs as biosensors toward disease diagnosis by using intracellular, stationary, and time-dependent information. Furthermore, the potential of machine learning (ML) and AI algorithms for developing biosensors can revolutionize any future outbreak.

## 1. Introduction

The quantum defects possessing extraordinary optical and electronic properties in fluorescent nanodiamonds (FNDs) owe a broad scope in the scientific horizon of biosensors for electromagnetic fields, including thermal and magnetic signals. Recent trends have novelties in surface modification and conjugation with biocompatible materials enabling the detection of static and time-dependent fields for ultrasensitive in vivo measurements. These efforts also allow the quantitative prediction of intracellular thermodynamics. Herein, we compile thermal and magnetic-sensing methodologies for rapidly detecting infectious viruses (viz., SARS-CoV, HIV, Ebola, and influenza) and deadly diseases (viz., primary and secondary cancers, Alzheimer’s, and Parkinson’s disabilities). We also assessed the feasibility of using FNDs as a therapeutic agent and wide-field microscopy for secondary-stage cancer, consequently improving public health. Further, the application of artificial intelligence (AI) in clinical decision making is vital for early disease diagnosis using different machine- and representation-learning strategies.

FNDs are nanomaterials carrying atomic-scale fluorescent defects, delivering direct applications in biosensing and bioimaging. These nanomaterials are beneficial compared to similar nanosensors, such as quantum dots and organic dyes, because of a higher quantum yield, longer photostability, and lower toxicity, mainly when utilized under ambient conditions [1]. The composition of the surface chemistry of FNDs allows functionalization with different groups of carbon because favorable covalent bonding leads toward enhanced photophysical properties enabling direct measurements in biological species [2]. In vivo biosensing is an emerging field that allows us to sense the biological activity of different cells and tissues. In this direction, various techniques are available, such as ultrasound imaging, magnetic resonance imaging, and X-ray tomography. Nevertheless, these approaches cannot deliver a nanoscale spatial resolution and high-sensitivity measurements under ambient conditions. The naturally occurring quantum defects in diamonds have demonstrated excellent results for sensitivity and spatial resolution at the nanoscale. Recently, FNDs have gained wide attention in biomedical research due to their superior biocompatibility, low toxicity, and direct implications in chemotherapy, as a drug delivery agent, without any prolonged harmful effects.

Nanodiamonds can be easily synthesized from a bulk material by thin films, nanorods, and nanoparticles. Single crystals in a millimeter size occupying fluorescent defects along preferred crystallographic planes can be fabricated with the chemical vapor deposition (CVD) method [3]. The CVD method has shown lower paramagnetic impurity content enabling an enhanced magnetic field sensitivity in the nanotesla regime [4]. This sample type generally provides a much better spatial resolution for imaging using individual defects than nanocrystals. Detonation nanodiamonds (DNDs) can be formed by detonating explosive compounds (TNT and RDX) in a controlled environment. As a result of the explosion, the supersaturated carbon vapors condense into tiny droplets, which later form nanocrystals. The DNDs available as ultra-small nanocrystals (less than 10 nm) are not considered favorable candidates for biosensing because of the low photo stability and impurities.

Diamond nanocrystals can be formed by crushing and grinding micron-size diamonds. Afterward, the high-pressure high-temperature method (HPHT) favors the formation of stable luminescent defects. The synthesis of small nanoparticles (1–5 nm) and large 100–500 nm particles can be accomplished using the HPHT method [5]. The naturally occurring impurity atoms in diamond are nitrogen (about 1% by mass), silicon, germanium, and tin. The luminescence emission of these defects is observed to have enhanced effects by increasing the nitrogen content to different extents of <80 and >372 ppm [6]. On average, the available nitrogen content in the HPHT and CVD-grown diamonds is 100 and 1 ppm, respectively. The presence of nuclear spins (^13^C, ^14^N, and ^15^N) interacting with the desired quantum defects in diamond have also been observed [7,8].

The nitrogen vacancy center (NV center), among the most commonly observed and studied luminescent defects in diamonds, is formed due to the combination of a nitrogen atom with a vacant diamond lattice site. The formation of the NV center in a diamond can be accomplished by ion implantation followed by annealing. The creation of NV centers in diamonds is observed to have a strong correlation with the energy of the ion implantation, causing the formation of shallow and deep defects [9,10]. The creation efficiency and depth of NV centers are (1%, 8 nm) for 5 keV ion energy and (45% micro-meter depth) for 18 MeV [11].

This review article encompasses the recent trends and advancements in biosensing applications with FNDs following ultrasensitive disease and virus detection using the electronic spin-state fluorescence of the NV center. The salient features of this review can be summarized as NV centers in surface-enhanced FNDs (100 nm) attached with Gadolinium complex magnetic nanoparticles which allow us the capability to detect the SARS-CoV virus (<100 RNA copies) in just one second with a low false negative rate (<1%). Size-controlled FNDs have been implemented as an efficient chemotherapy drug delivery agent and enhance the drug uptake in glioma cells (brain), thereby improving the effectiveness of chemotherapy medicines. Moreover, the NV spin relaxation in cellular media has also monitored tumor growth up to 1 mm. The structure of this article is arranged so that Section 2 describes the basic principles and state-of-the-art achievements in nanothermometry. A comprehensive summary of intracellular metabolic heat generation and the effects of magnetic hyperthermia are also included. Section 3 focuses on magnetic microscopy and tracking with FNDs, starting with the basic principles followed by intracellular applications. Section 4 introduces a collection of recent highlights of intracellular quantum sensing for neural activity imaging and high-resolution NMR spectroscopy. Section 5 combines the biosensing applications of FNDs for infectious virus and disease detection. Section 6 summarizes the possibilities and outcomes of using AI strategies recommended for early stage disease diagnosis and imaging. Section 7 elaborates on the futuristic prospects, ongoing challenges, and limitations of the significant research in biosensing. Section 8 concludes the essential findings and recommendations of this work.

## 2. Intracellular Heat Generation and Thermal Sensing

This section describes the basic principle of NV nanothermometry and the consequential outcomes of optical spectroscopy. Intracellular NV thermometry is quantitatively examined, highlighting the most recent and benchmark achievements. This section also includes a brief analysis of the reported data for the intracellular metabolic heat generation associated with magnetic hyperthermia.

### 2.1. Optical Nanothermometry

**Figure 1:**
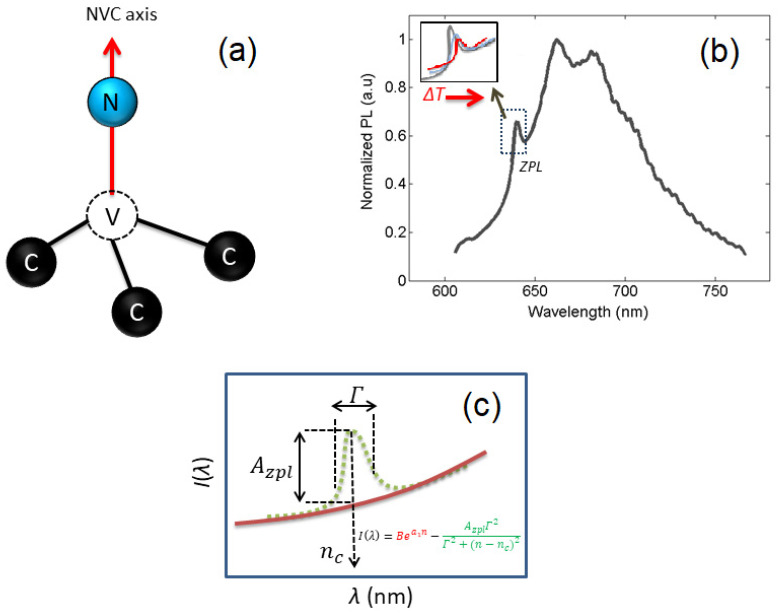
Optical spectroscopy of NV center. (**a**) The structure of the NV center in a diamond comprising a nitrogen atom connected to a vacancy (center). The NV center is optically excited typically with 590–532 nm lasers, whereas the emission lies within 500–800 nm. (**b**) PL spectra of a single NV center displaying ZPL (638 nm) and PSB; inset shows the temperature-dependent shift in ZPL. (**c**) The mathematical representation of the shape of ZPL using a Lorentzian function with an exponential background.

The atomic structure of the NV center appears as an artificially trapped molecule in a diamond cubic, Figure 1a, exhibiting distinct optical and electronic properties suitable for nanoscale thermometry [12,13,14], magnetometry [15,16,17,18], electrometry [19,20], pressure sensing [21,22], and as fundamental charge detection [23]. The NV center in diamonds has been observed in various charge states (NV^0^, NV^+^, and NV^−^), although the negatively charged NV^−^ center possessing extraordinary properties at room temperature has been extensively used for nanosensing purposes over the past two decades [24,25]. The absorption and emission spectra of the NV^−^ center lies within the visible range of the electromagnetic spectrum; therefore, easily accessible optical lasers (532 nm) can be used for excitation. The emission spectra of the NV^−^ center comprise a Zero-Phonon Line (ZPL) at 638 nm along with broad phonon side-bands (PSB), Figure 1b. In the NV^−^ center’s photoluminescence (PL) spectra, just a few percent of the luminescence fall inside the ZPL, whereas the rest of the photons contribute to the formation of the PSB due to the presence of thermally excited vibrational states. The fluorescence lifetime of an NV center is measured to be within 10–12 ns [26].

The key feature of the PL spectra of an NV center is the dependence of the ZPL on the temperature and magnetic field-dependent fluorescence intensity whenever subject to resonant microwave excitation. Under resonant excitation, the NV center population can be shifted toward the available degenerate spin sublevels, followed by relaxation through the singlet states (intersystem crossing), alleviating the luminescence emission (30%) [1]. The detection of the NV luminescence under sweeping radio frequency signals is termed the optically detected magnetic resonance spectrum (ODMR). The ODMR method is a primary tool for nanoscale DC magnetometry [27]. However, pulsed schemes have also been devised to detect AC fields. Moreover, the zero-field ODMR spectra are also susceptible to thermal changes due to electron–phonon interactions and the thermal expansion of the lattice, which have been widely investigated to have a temperature-dependent shift (−74 kHz/K) for 280–330 K [28]. Besides the ODMR thermometry, a much simpler all-optical method is also available [29,30], enabling us to achieve a thermal noise floor comparable with the CW-ODMR technique [31].

The thermally induced shift in the position of the *ZPL* at room temperature has been determined as $\frac{d\;ZPL}{dT}=0.015\;\mathrm{nm}/K$ [32,33]. The all-optical ratiometric thermometry relies upon the ratio of the counts underneath the *ZPL* and the total emission spectra, known as the Optical Debye–Waller factor. The experimental implementation of the all-optical method requires the acquisition of the photoluminescence spectra of NV centers at different temperatures. The thermal shift of the *ZPL* is illustrated in Figure 1b. The *ZPL* has been represented as a Lorentzian function with an exponential background, as illustrated in Figure 1c. The experimental PL spectra can be analyzed for the estimation of the position, amplitude, and width of the *ZPL* by minimizing the error function [31], as given by

(1) $$\chi^{2}=\sum_{n}{\left(N_{n}-Be^{a1n}-\frac{A_{Z}\Gamma^{2}}{\Gamma^{2}+{\left(n-n_{c}\right)}^{2}}\right)}^{2},$$

where *A_z_*, Γ, and *n_c_* correspond to the amplitude, width, and position of the *ZPL*. *N_n_* shows the detected photons at the *nth* pixel of the CCD and *B*, *a*_1_ are the fitting parameters for the exponential background. The all-optical method using 590 nm excitation has shown a temperature noise floor of 0.3 KHz^−1/2^, employing ~50 nm nanodiamonds [31]. The implementation of this method with SiV centers in nanodiamonds (<250 nm) has achieved a thermal noise floor of 13 mKHz^−1/2^, leading to the detection of 0.4 °C thermal fluctuations in 1 ms [34]. Hybrid nanothermometers were introduced recently, where a magnetic nanoparticle is placed at a fixed distance from a nanodiamond, enabling ultrasensitive nanothermometry. This enhances the thermal susceptibility of the NV spin resonance by two orders of magnitude [35,36]. Hence, the induced change in the temperature is transformed into the magnetic field strength of the attached magnetic nanoparticle near its Curie temperature via the nanodiamond sensor. A thermal sensitivity of 76 µKHz^−1/2^ has been demonstrated along with observing a 2 mK thermal variation with a time resolution of 5 ms [35]. Besides the optical and NV spin resonance-based methods, the possibility of using NV center-excited state lifetimes for thermometry has also been explored [37]. This technique employs a single photon counting setup (300–500 K) and pulsed excitations for lifetime measurements that provide a temporal and spatial resolution of nearly 100 ns and 100 nm, respectively.

### 2.2. Intracellular Thermometry

The observation of the microscopic temperature is a challenging step toward the understanding of complex living biological systems. Ideally, a biosensor is required in this scenario, which can be easily integrated with biological species and enable the optical–thermal readout in real-time for accurate measurements of the intracellular metabolism. The follow-up details in this section summarize the efforts toward non-invasive intracellular thermometry. Kucsko et al. [14] introduced the idea of using gold nanoparticles (~100 nm) for localized heating upon 532 nm laser exposure in live human embryonic fibroblasts and reported a thermal sensitivity of 9 mK Hz^−1/2^ along with a sub-micron spatial resolution. In this experiment, the cellular metabolic activity was observed until cell death was confirmed. Gold nanorods conjugated with FNDs injected in humans showed controlled hyperthermia in embryonic kidney cellular membranes [38]. In this work, gold nanorods worked as heaters by laser-induced heating. Hence, a temperature change (28–75 °C) was observed using a thermally induced shift in the ZPL position.

A detailed study regarding in vivo thermometry in *Caenorhabditis elegans* live worms was reported using polyglycerol-coated nanodiamonds (181 nm) [39]. A chemical stimulus was injected into the live worms, which produced localized heating upon optical illumination, resulting in detectable heat generation. CW-ODMR spectroscopy was implemented to achieve a thermal sensitivity (1.4 K Hz^−1/2^) with a precision of 0.2 K. Another study explored the FND-Polydopamine (FND-PDA) as a nano heater-thermometer, where the thickness of the Polydopamine layer (5–30 nm) coated on 100 nm FNDs was selected for the optically controlled localized heating and thermal sensing [40]. The FND-PDA composite biosensor material was then employed for the experimental evaluation of HeLa cells’ thermal conductivities and heat exchange (0.1 nJ) during cellular metabolic reactions for a less than 50 µs time interval [41]. Similar studies confirmed that composite materials based on FNDs are potential candidates for the analysis of nanoscopic thermodynamics [36]. Fiber-probed all-optical thermometry using germanium-vacancy centers in FNDs were tested on live mice for real-time brain temperature sensing [42]. A micron-size FND attached at the tip of a fiber probe enabled the observation of a thermal variation of 0.1 °C using the thermal shift in the PL emission spectra.

### 2.3. Quantifying the Intracellular Metabolic Heat

This section aims to assess the feasibility of FND-based thermometry for observing intracellular metabolic heat. Here, we compile the details of in vivo studies using conventional techniques for intracellular heat generation in various cellular reactions. The thermometric data included here could become a roadmap for FND-based investigations of intracellular biochemical reactions, including protein synthesis, cellular respiration, thermogenesis, and abnormal heat generation in infected cells.

A recent study has shown that the mitochondria could maintain a stable temperature at 50 °C during the cellular metabolism, which is significantly larger than the cytoplasm and cellular membrane [43]. It is observed that the released energy is consumed to synthesize adenosine triphosphate during intracellular respiration. The experiments with live human embryonic kidney cells (HEK-293) and skin fibroblasts revealed that the mitochondrial temperature was 10 °C higher than the ambient temperature. The results also indicate a maximal activity of Respiratory-Chain enzymes at 50 °C. Wang et al. described using a thermocouple probe for the in vivo thermometry of U251 cells [44]. The addition of the chemotherapy drug (Camptothecin) to the U251 cells rapidly enhanced the intracellular temperature by 0.5 °C, approaching a maximum value of 1 °C. Magnetic hyperthermia (MH) is a clinically tested biocompatible technique in chemotherapy and radiotherapy for generating controlled heat by using magnetic nanoparticles under the influence of alternating magnetic fields. MH-induced cellular apoptosis in DX3 human melanoma cells has been demonstrated at the maximal heating of 51 °C. The cell death process was followed up using Annexin (V-FITC) for the initial and permanent apoptosis. The percentage of detected live cells over two hours of treatment lies within 35–91%.

## 3. FNDs in Magnetic Sensing and Microscopy

This section presents the basic principle and theoretical background of magnetometry using NV centers in FNDs and its applications in imaging and tracking in biological media. The experimental setup generally employed for wide-field magnetic imaging is described along with the data collection based on the best-achieved sensitivities tabulated. A comparison of the photophysical properties of NV centers with similar optical probes (organic dye and quantum dot) is also included.

### 3.1. Optical Magnetometry

**Figure 2:**
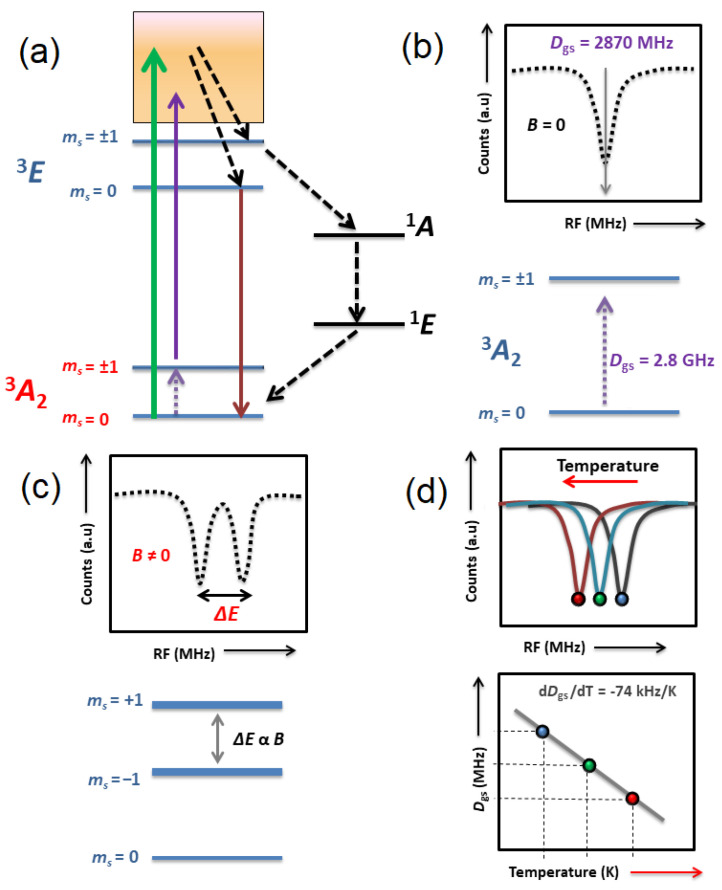
Electronic structure of NV center and magnetic interaction. (**a**) Schematic of the energy levels of NV center, depicting optical excitation and relaxation. Intersystem crossing through singlets states (^1^*A*,^1^*E*) is shown in black arrows and the resonant microwave excitation is purple. (**b**) The ODMR spectra at zero field (*B* = 0). (**c**) The ODMR spectra at (*B* ≠ 0) displaying the Zeeman splitting between the spin sublevels. (**d**) Thermally induced shift in the position of *D_gs_*.

The NV center electronic structure reveals that both the ground and the excited states (^3^*A*_2_ and E^3^) are composed of spin-triplet states (m_s_ = 0 and m_s_ = ±1) as depicted in Figure 2a. The NV center population can be accumulated in the spin sublevel (m_s_ = 0) upon optical excitation. In contrast, the spin sublevels (m_s_ = ±1) overlap each other under a zero external field, the ground-state zero-field splitting (ZFS), of 2.8 GHz, as illustrated in Figure 2b. In this situation, a resonant microwave excitation can transfer the NV center population from m_s_ = 0 to m_s_ = ±1, which decays back to (m_s_ = 0) due to the available singlet states [45,46]. The population decay through this channel causes a drop in the NV center luminescence emission up to 15–20%, which is widely observed in lines in the ODMR spectrum [47]. This phenomenon can be observed under zero and non-zero external magnetic fields, as shown in Figure 2b,c. The zero-field ODMR line *D*_gs_ arising at 2.8 GHz is very sensitive to thermal fluctuations and a direct tool for thermometry, as shown in Figure 2d [28]. In the case of a non-zero magnetic field pointing along the NV center axis, a pair of lines appear in the ODMR spectra (Figure 2c), where the energy splitting between the lines is directly dependent upon the magnitude of the magnetic field as predicted by the Zeeman effect. The interaction between the NV center and arbitrarily oriented magnetic fields can be incorporated in the form of a ground-state spin Hamiltonian as given by [48,49,50]

(2) $$\hat{H}=hD_{gs}{\hat{S}}_{z}^{2}+hE\left({\hat{S}}_{x}^{2}-{\hat{S}}_{y}^{2}\right)+\gamma_{e}\vec{B}\cdot \hat{S}$$

where *E*, $\gamma_{e}$, and $\vec{B}$ are the strain-induced splitting, NV electronic gyromagnetic ratio (28 MHz/mT), and the magnetic field vector. ${\hat{S}}_{x},\;{\hat{S}}_{y},{\hat{S}}_{z}$ are the spin-1 operators. In the case of a single NV center, if the magnetic field is directed along the NV axis, then the spin Hamiltonian can be solved for an unknown magnetic field using approximation as $B=\Delta F/\left(2\gamma_{e}\right)$, where ΔF is the energy spitting between the two lines in the ODMR spectrum.

A three-dimensional vector field can be reconstructed using at least three or four differently orientated NV centers [51,52,53]. Magnetometry using an ensemble of NV centers (40 nm FNDs) along four different orientations has shown that fields within the range of 10–20 mT can be accurately determined down to 20 µT [52]. The sensitivity of the ODMR technique can be enhanced by either reducing the width of the ODMR lines or increasing the number of emitters in the FNDs. The NV centers in FNDs have been widely used as a single ensemble of sensors for nano-magnetometry, demonstrating a sensitivity in the pico-tesla regime [15].

### 3.2. Magnetometry in Biological Species

**Figure 3:**
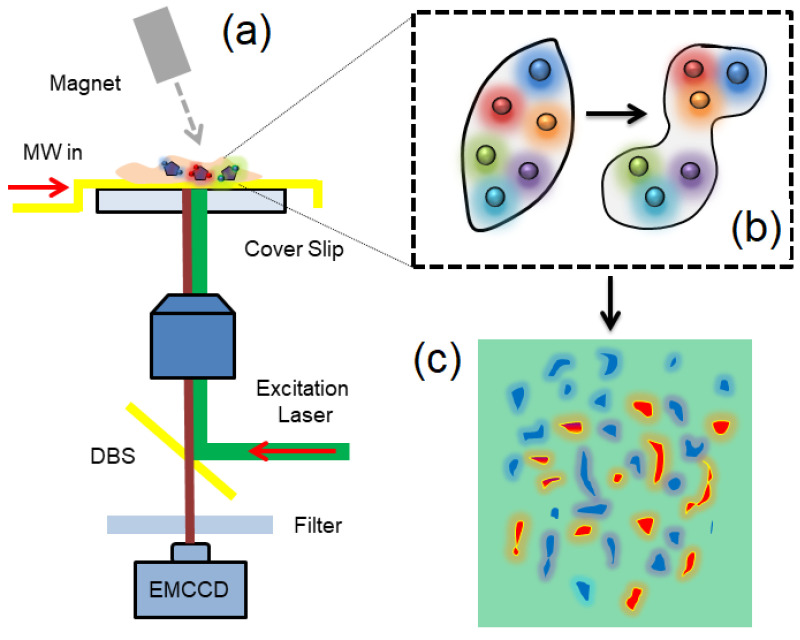
Wide-field magnetic imaging with FNDs. (**a**) Optical spin microscope based on the luminescence of NV centers in FNDs for bioimaging. FNDs are spin coated on a glass coverslip and printed with gold electrodes for microwave signal transmission. The microscope comprises objective, dichroic mirror (DM), filter, and permanent magnet. Fluorescence images are recorded by EMCCD while the sample is excited with a 532 nm laser and a scanning microwave frequency source. (**b**) Schematic of surface-modified FNDs for bio-labeling and tracking. (**c**) The reconstructed magnetic image displaying the presence of labeled magnetic organisms.

This section accumulates the details relevant to intracellular magnetic sensing using FNDs. The setup employed for intracellular and biological sensing is depicted in Figure 3a. A tabulated form of reported sensitivities, along with sensing protocols, is shown in Table 1. An example of magnetic imaging in living organisms (magnetotactic bacteria) has been demonstrated in Figure 3c when iron-oxide magnetic particles (50 nm) were injected into living worms [16]. The magnetic particles formed chains inside the living organism, which was detected (0.5 Gauss) and imaged using wide-field microscopy (400 nm spatial resolution) employing CW-ODMR.

The ODMR scheme, widely used for magnetic sensing, is a common choice for imaging biological species, such as chicken breast tissues and neural stem cells [51,52]. FNDs embedded in polymer nano-fibers enabled the realization of neural activity over a millisecond temporal resolution and 3.4 µT Hz^−1/2^ sensitivity. Another experiment demonstrating superresolution imaging in human breast cells (MCF10-A) was conducted by employing 20 nm iron-oxide magnetic nanoparticles tagged with 70 nm FNDs [53]. The measurements of the composite sensor magnetic nanoparticles FND reported a 100 nT Hz^−1/2^ field sensitivity with a 20 nm spatial resolution. The target cells labeled with FNDs were easily distinguishable from the normal cells, providing a much broader scope of this method for enhanced biosensing and bioimaging compared to conventional optical microscopy.

### 3.3. Intracellular Imaging and Tracking

FNDs, due to the availability of different sizes, the concentration of NV centers, and compatibility with optical microscopes, are ideal for sensing and tracking the intracellular environment, especially with live cells [54]. The NV centers luminescence in different FNDs (5–100 nm) has been utilized for monitoring the cellular division in the Paramecium caudatum and Tetrahymena thermophile living microorganisms, as shown in Figure 3b [55]. The live microorganisms were injected with 5 nm FNDs and kept under observation for two days. Afterward, it was found that the growth rate of T. Thermophile was reduced to 50%, whereas the growth of P. Caudantum was terminated. The in vivo measurements revealed that the microorganisms could store the FNDs (100 nm) in food vacuoles, and later on, the excretion was also observed. In this experiment, the 5 nm FNDs were more effective in reducing cell growth. Overall, this experiment explored the possibility of using FNDs for the accumulation of experimental data regarding the digestive system of microorganisms, which can further lead to the development of a theoretical model for food intake, cell growth, and death.

### 3.4. Superresolution Microscopy

**Figure 4:**
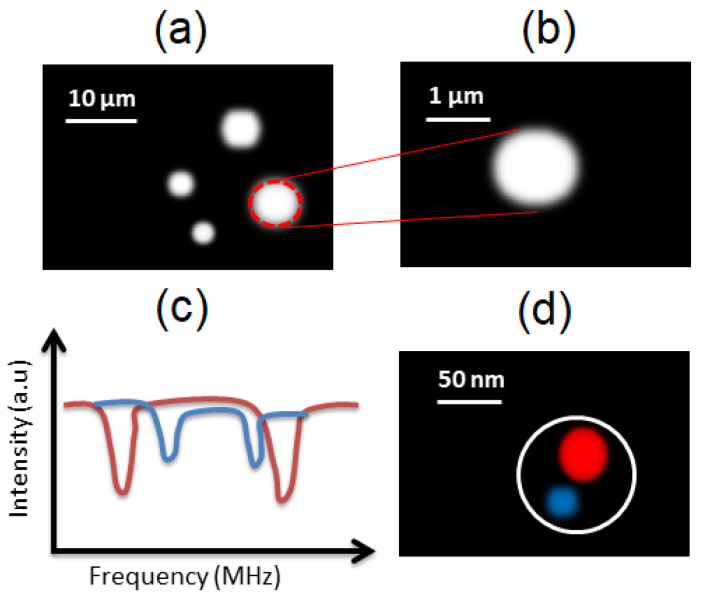
Illustration of the multispectral superresolution microscopy using NV centers. (**a**) FNDs shown as arbitrarily dispersed bright spots. (**b**) Single FND illustrated in large view. (**c**) The ODMR spectra of single FND, as in (**b**), displaying two differently orientated NV centers. (**d**) The reconstructed superresolution image indicating red and blue spots as two different NV centers.

The applications of NV centers in FNDs for imaging have now been extended toward superresolution microscopy, which could be further stretched to explore intracellular structures and the associated biochemical reactions at the molecular level (Figure 4a,b). The fluorescent imaging with FNDs can easily be integrated with advanced microscopy techniques such as confocal, Raman, and structured illumination microscopy. The long-term photostability and exceptional sensing capability of NV centers with a nanometer resolution are advantageous compared to several bioimaging counterparts, such as fluorescent dyes and quantum dots. A comparison between these three bioimaging agents is shown in Table 2.

Imaging complex molecular structures beyond the diffraction limit is a crucial step toward understanding the molecular structure of proteins, lipids, and subcellular organelles. In this direction, the magnetic resonance of closely lying (<50 nm) NV centers has been used to deterministically control the luminescence emission of an individual NV center in a technique called multispectral superresolution imaging, enabling a spatial resolution down to 12 nm and resulting in the identification of single NV centers in a 1.4 s measurement time as shown in Figure 4c,d [56]. This technique is based on forming magnetic field gradients in concise domains (<50 nm) where the NV center spin-dependent luminescence can be switched by carefully selecting the resonant microwave frequency. The analysis of the point spread function of the diffraction-limited bright spots using Gaussian fitting allowed to optically resolve several single NV centers distributed with an FWHM span of 12–45 nm.

## 4. Intracellular Quantum-Sensing Applications of Surface-Enhanced FNDs

This section incorporates a collection of recent highlights of intracellular quantum-sensing applications of surface-enhanced FNDs. These modified fluorescent nanosensors appear appropriate for subcellular organelles involving a wide range of intracellular research, including PH sensors, free radicals, analyte concentration, neuron activity, and ultrasensitive nuclear magnetic resonance spectroscopy.

Functionalized nanodiamonds (PEG-FND) play their role in labeling the cellular membrane, which could further be utilized for immunostaining, flow cytometry, and intracellular signaling. The interaction between the nanodiamonds’ surface and biomolecules is critical before moving on to initiate biomedical research. A group of proteins and salts can easily bind with an FND surface presenting good biocompatibility and cellular uptake [57]. In addition, it is realized that the shape and size of FNDs influence the trafficking in cells, inferring that the smallest particles are mostly dispersed in the cytoplasm. In contrast, the others form aggregates in endocytic vesicles [58].

The bio-membrane is a protective biological shield for regular embolic activity while preventing the cell and its organelles from external hazardous species and infections. Fluctuations in the activation potential of neurons are instigated during inter-neuron communication, producing weak magnetic signals. In this regard, NV magnetic sensing has been used as a potential tool for monitoring endogenous neuronal activity for the high-resolution mapping of intracellular magnetic signals [59]. The multi-electrode approach in primary cortical neurons has confirmed the nontoxic consequences of FND uptake and allowed magnetic and thermal sensing to evaluate the neural network spike amplitude and burst rate. In addition, this strategy also enabled neural thermometry with a sensitivity of 1K [60].

A similar study has shown the detection of inter-lipid magnetic signaling within an artificial lipid bilayer [61]. The NV spin coherence measurement has been used to observe ion motions through ion channels [62]. Mitochondria are considered a rich source of production of free radicals during triphosphate synthesis. The conventional methods of free radical detection rely on fluorescent dye, which unfortunately suffers from photostability and reverses the reaction. FNDs coated with surface-adsorbed VDAC2 antibodies were subject to being near mitochondria [63]. NV spin relaxometry conducted on the mitochondrial surface of macrophage cells enables the detection of magnetic noise, which is attributed to the free radicals’ production (CCP) within the range (1–10) µM.

Furthermore, the NV spin relaxometry using multiple pulse sequences has been utilized to differentiate and detect various free radicals, perfluoropolyether analyte concentrations, and chemical shifts [64,65]. Ultrasensitive NMR spectroscopy carried out using NV spins in diamond has shown the possibility to attain a spectral resolution that is enough to discriminate between the alkyl group and vinyl group by sensing individual spins. The sensing spin protocols were optimized to achieve the maximum NMR sensitivity to sufficiently detect chemical shifts (^1^H and ^19^F) in a 20-zeptoliter volume [65]. A plasmon-enhanced FND nano-assembly has also been introduced for imaging and biosensing in HeLa cells [66]. When subject to a proper sensing methodology, functionalized nanodiamonds allow controlled intracellular heating (photothermal therapy) and thermometry in the HeLa cell line [67].

## 5. FNDs for the Detection of Infectious Viruses and Malicious Diseases

The human body is constantly threatened by harmful microorganisms, such as viruses and bacteria. These microorganisms invade the human immune system and replicate themselves by injecting genetic material into the cell nucleus, thus disrupting normal cellular functions. This phenomenon occurs at the cellular level, resulting in malfunction in the cellular metabolic activity and further leading to an increase in body temperature. According to the World Health Organization statistical database, approximately 650,000 people die every year due to respiratory diseases caused by the flu and infections. In contrast, the number of globally infected patients due to the coronavirus (COVID-19) extends to 619 million, causing 6.5 million deaths worldwide. Cancer is also one of the leading causes of global deaths annually, causing 10 million deaths in 2020. Every year, more than 9 million new cancer cases are diagnosed, and lung cancer contributes to 18% of deaths. Approximately 0.5–1 million people die from HIV/AIDS every year globally. It is generally accepted that the health damage caused by diseases such as HIV/AIDS, cancer, Alzheimer, and Asthma can be significantly reduced if diagnosed at early stages. One of the goals of the emerging research in biological sciences and nanotechnology is to develop novel strategies for the efficient, rapid, and accurate detection of viruses and diseases. In this section, we compile the results of the FND-based ultrasensitive biosensing of infectious viruses such as SARS-CoV-1 (namely SARS-CoV), HIV-1, Ebola, and influenza. We have discussed FNDs as an efficient therapeutic drug delivery agent for various cancers, viz., the brain, lung, and breast. FND microscopy has been implemented to monitor the morphology and growth of cancer cells. The intracellular response of NV magnetic sensing works well toward the presence of free radicals under viral infection.

### 5.1. Biosensing of Infectious Viruses

During the recent COVID-19 pandemic outbreak, faster and more reliable detection techniques are essential [68]. A rapid antigen test allows medical specialists to get the results within 1 day; however, the accuracy is compromised. In the case of a virus outbreak like COVID-19, the accuracy of tests is a significant issue because a false report might lead to an infected patient not being isolated, causing a large population under threat. The wide range of options for biocompatibility, lower toxicity, cellular uptake, cell viability, and stable fluorescence have enabled FNDs to be a very competitive biomarker and ultrasensitive biosensor under ambient conditions. The optical sensing using surface-modified FNDs conjugated with polymers, organic/inorganic molecules, magnetic nanoparticles, peptides, and amino acids allows highly sensitive, reliable, and faster measurements under clinical tests [69].

The electronic spin of the NV center in the diamond, an excellent quantum sensor, is primarily realized for quantum computing. However, during the past few years, atomic defects in FNDs have emerged as an ultrasensitive tool for biosensing at a molecular level [70,71]. Chang et al. utilized the unique magneto-optical properties of an NV^−^ center to develop a spin-enhanced lateral flow immunoassay (SELFIA) platform for an ultrasensitive biomedical analysis by using an FND as an alternative reporter [72]. The SELFIA-FND marker system can effectively remove the background fluorescence signals and obtain a lower detection limit than colloidal gold particles of a similar size. The selective and ultrasensitive detection of FND particles at a density of 0.04 ng/mm^2^ on the nitrocellulose membrane was used for SELFIA. The FND-based detection showed a 100-fold greater sensitivity compared to a nanogold-based lateral flow immunoassay (LFIA) and surpassed an enzyme-linked immunosorbent assay (ELISA) in terms of efficiency and flexibility. In addition, the utility and versatility of SELFIA were demonstrated and further investigated for detecting SARS-CoV-2 antigens. The highly sensitive detection of SARS-CoV-2 N and S antigens from different variants was achieved, with a detection limit of 1.94, 0.77, 1.14, 1.91, and 1.68 ng/mL for the N protein, wild-type, Alpha, Delta, and Omicron S proteins, respectively [73].

**Figure 5:**
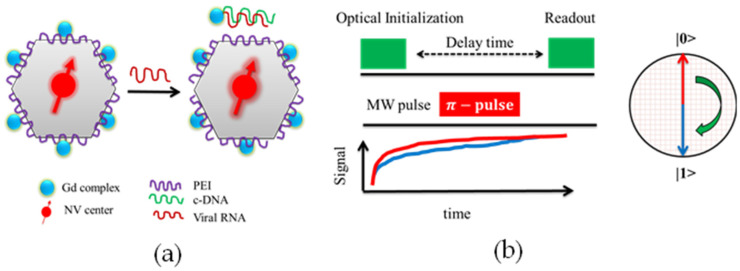
Schematic of SARS-CoV-2 detection using FND [75]. (**a**) Surface-modified FNDs which are coated with PEI are subject to binding with c-DNA-Gd^3+^ complexes. When these modified FNDs are exposed to virus RNA, the c-DNA-Gd^3+^ molecules covalently bond with the virus RNA and detach from the surface of the FND, resulting in a lower magnetic noise sensed by the NV center. The change in magnetic noise sensed by NV center can be observed in terms of NV luminescence emission. (**b**) The sequence of optical metrology using FNDs for T_1_ relaxometry.

The sensitivity of measurements has been improved to such an extent that a single protein and DNA can be detected using the characteristic electronic spin resonance signature [74]. Single NV centers in functionalized nanodiamonds (25 nm), which were PEI (Polyethyleneimine) polymer-coated, enabling the surface adsorption of complementary DNA (cDNA), as illustrated in Figure 5a, chemically bound with Gd^3+^ complex magnetic molecules, are capable of optically detecting SARS-CoV-2 RNA copies of less than 100 in 1 s of integrated time [75]. In the presence of the SARS-CoV virus RNA, the c-DNA-Gd^3+^ will be detached from the FND surface and bound with the virus RNA. Consequently, the NV center inside the FND will be under a reduced magnetic influence of the remaining surface-attached Gd^3+^ complex molecules. The magnetic noise of the Gd^3+^ molecules lying close to the NV center can be optically determined by their respective T_1_ relaxation time. The measured T_1_ will be longer than the initially measured T_1_ relaxation time under the presence of virus RNA. The measured T_1_ follows an exponentially decaying curve as a function of the surface density of Gd^3+^ magnetic molecules. The proposed optical biosensing method has shown a false negative rate of 1%, enabling a rapid and reliable testing platform. The magnetic noise strongly influences the T_1_ (spin-lattice relaxation) in the vicinity of the NV center. A schematic of the optical-sensing protocol is shown in Figure 5b, where the luminescence intensity of optically addressed bright and dark states was collected by applying a time-varying pulsed RF signal near the resonance frequency of the NV center. The change in magnetic noise in the vicinity of the NV center can be expressed as a stronger fluorescence intensity detectable by using conventional optical microscopes, thus identifying the number of SARS-CoV RNA in the respective environment. The sensitivity of SARS-CoV virus RNA detection using FNDs is strongly dependent on the fluorescence emission of the NV center but limited by the magnetic spin interaction with neighboring NV centers and magnetic impurities. Therefore, a single NV center in high-purity diamond crystals will be preferred for the measurements.

Human Immunodeficiency Virus (HIV) is another deadly virus that invades the hu-man immune system and weakens the ability to fight against infections causing acquired immune deficiency syndrome (AIDS). Clinically there are three tests for HIV/AIDS: the antibody, antigen, and nucleic acid tests. The antibody test, a rapid test, finds the concentration of antibodies in oral and blood samples. The antigen test involves blood samples from a vein and finds the number of antigens/antibodies. The nucleic acid test depends on the number of virus RNA in the bloodstream.

Fluorescence-based biomarkers are capable of sensitive detection in biological media [76]. Still, the sensitivity is limited due to the cellular autofluorescence signal and the background from the sample substrate. Conventional drugs using combination antiretroviral therapy (CART) for treating HIV-1 may induce neurological disorders, such as depression, anxiety, mental confusion, and forgetfulness. Nanoparticles for efficient drug delivery have tremendous implications in the field of diagnosis and therapy. Surface-enhanced nanoparticles are now capable of combining and transporting a variety of nanomedicines toward the target sites. A challenging problem is the efficient transport of therapeutic medicines toward HIV-infected organs such as the central nervous system, primarily due to the lower transmigration of treatment through the blood–brain barrier. Recent efforts to enhance the efficacy of nanomedicine drug delivery have brought FNDs into this framework, a carbon-based nanomaterial exhibiting high biocompatibility and lower toxicity in biological media. Surface-modified FNDs with -COOH and -NH_2_ groups were tested with anti-HIV-1 medicine (Efavirenz) for loading capacity and toxicity [77]. FNDs allow surface modification for binding with various functional groups, such as the carboxylic group, hydroxyl group, lactone, ketone, and ether. It is concluded that unmodified FNDs, when conjugated with HIV-1 nanomedicine, demonstrated a higher loading capacity and lower toxicity than surface-modified FNDs. Hence, FNDs offer a superior performance for enhanced drug delivery to the central nervous system [77]. FNDs combined with a microfluidic lateral flow assay (LFA) and magnetic modulation have shown the potential of being a stable biomarker exhibiting high brightness for in vitro diagnostics of HIV-RNA [78].

**Figure 6:**
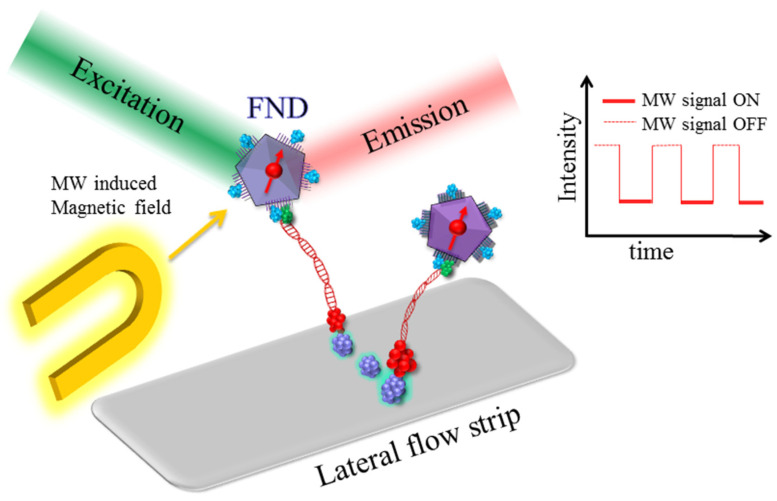
Schematic of the principle of HIV-1 RNA detection using FNDs and RT-RPA on the LFA. Labeled amplicons were generated during the reaction between Digoxigenin (DIG) and biotin-modified primers. The DNA primer binds with the HIV-1 virus RNA which is extracted from the patient. The primer then binds with c-DNA followed by purification and amplification. The labeled amplicons specifically bind with the functionalized nanodiamonds (FND-anti-DIG)) and streptavidin (purple) being immobilized on the lateral flow test strip. The NV center luminescence is recorded by applying microwave (MW) pulsed sequences in ON and OFF states.

Functionalized nanodiamonds conjugated with antibodies by disuccinimidyl carbonate (DSC) trigger the hydroxyl surface group forming succinimidyl carbonate and enabling it to rejoin with antibodies to develop stable carbamates. Three different sizes of FNDs, viz., 120, 200, and 600 nm, were implemented for antibody functionalization, whereas Digoxigenin (DIG) and biotin primers allowed RT-RPA amplification, allowing the binding with test lines (the main notion derived from [78], Figure 6). This method applies the RT-RPA for HIV RNA detection. The RPA reaction was conducted using 2 µL of target HIV-1 RNA at 37 °C for 10 min. The reported limit of detection using 120, 200, and 600 nm FNDs is (200 aM, 46 aM, and 820 zM), respectively. This study provides a better correlation with large particle sizes of FNDs due to a greater fluorescence intensity and signal-to-noise ratio. The microwave-assisted spin-dependent fluorescence of the NV center in a large FND (600 nm) allowed the enhanced sensitivity for a single copy of HIV-1 RNA detection in 10 min of RTRPA processing. The LFA strip is initially printed with the non-specific binding of streptavidin after washing the test strip with milk (non-fat). The immobilized FNDs attracted to the test strip were excited by a 550 nm LED followed by imaging with an optical microscope attached to a camera. The magnetic field-induced NV fluorescence was recorded under the effect of an amplitude-modulated RF pulse sequence along with a lock-in algorithm for a frequency-domain analysis.

Normal biochemical reactions govern the abundance of free radicals in the human body. The viral infection generally modifies the intracellular concentration of free radicals. The precise monitoring of free radicals can allow infectious viral detection at the intracellular stage. Magnetic sensing using an FND has been applied to observe the response of free radicals in baby hamster kidney cells under the invasion of Semliki forest virus infection [79]. The intracellular measurements of the T_1_ relaxation time enable the observation of free radicals during virus replication. The study provides an opportunity for real-time observations of the cytoplasmic transport at an early stage of viral infection. The in vitro and in vivo transport of FNDs encapsulating Cowpea chlorotic mottle virus proteins in live HeLa cells was reported [80]. The strong NV luminesce enables live 3D tracking and could be utilized to understand the viral interaction with cellular organelles (endocytosis).

Ebola is a deadly virus that spreads through the interaction between animals and humans and originates from bats due to an outbreak in West Africa. It is believed that the viral infection has not been eliminated, so the outbreak remains regular. To cope with this problem, the major obstacle is the unavailability of rapid, sensitive, and accurate diagnostic tools. Fluorescent biomarkers such as organic fluorophores, semiconductor quantum dots, and fluorescent dyes are considered suitable candidates, depending upon their biocompatibility. However, photo-blinking, photo-bleaching, and poor long-term stability are the drawbacks of these available tools. In contrast, NV centers in FNDs exhibit the ideal properties of being a suitable biomarker. Efforts have been carried out to enhance the optical properties of FNDs tailored according to the requirements of the near-infrared emission. These modified FNDP-NV particles were reported in [81]. The method utilized an LFA scheme implementing NV centers in nanodiamonds for Ebolavirus detection. The FNDP-NV (200–800 nm) were connected with anti-Ebola (Glycoprotein) monoclonal antibodies for reacting against the Ebolavirus and were then tested over an LFA scheme along with recombinant-GP-cultivated monoclonal antibodies c13C6. The 200 nm FNDP-NV-c13C6 were attached with Ebolavirus glycoprotein and successfully captured at the test line by monoclonal antibodies 6D8. The NIR luminescence emission verified the capture of FNDP-NV-c13C6. The FNDP-NV reporter for EBOV-GP provides an opportunity for the rapid diagnosis of the Ebolavirus.

Influenza is another viral infection that attacks the respiratory system through the nose and throat, usually appearing as the flu in winter. Influenza flu has several types. Type A and type B cause seasonal flu symptoms. The type A influenza virus is known as the flu pandemic. The rapid antigen test can be conducted to identify viral genetic material using a nasal swab or saliva sample from the patient processed through an RT-PCR test, giving the results within one day. A chemical interaction between diamond (amorphous powder) and influenza virus type A and type B have been examined [82]. It has been observed that the virus c-DNA (200–400 µL) can be effectively adsorbed by the FND (4 mg) sorbents which can be further segregated and dissolved in an aqueous solution in just 20 min under ambient conditions, giving up the opportunity to develop an FNDs-based viral filter and control setup.

### 5.2. FND as Nanomedicine for Cancer Therapy and Disease Diagnosis

The optical detection of brain tumors using FNDs has emerged as an appealing tool due to its excellent biocompatibility, low cytotoxicity, and photostability, enabling long-term particle tracking, cell labeling, and imaging [75]. In the framework of intracellular biosensing, we have included the details of FNDs as a therapeutic drug delivery agent, monitoring the growth of a tumor and controlling cancer cell death.

Surface-modified FNDs with PEGylated denatured Bovine-serum albumin (BSA) and tumor vasculature-pursuing tripeptides were utilized for suppressing the in vitro blood–brain barrier (BBB) permeability and effectively approaching the target site [83]. The BBB is a significant barrier to the delivery of drugs used for diagnosis when the particle size exceeds 200 nm. Therefore, surface modification is essential for drug penetration. The mechanism of endocytosis relies on receptor binding at the endothelial cell surface. This transport can be facilitated with size-controlled and surface-enhanced nanoparticles [84]. Protein-coated FNDs exhibit a strong colloidal stability in solvents and reduce the formation of aggregates [85,86], whereas carboxyl-FNDs tend to form clusters [87]. The respective T_2_ weighted images can monitor brain tumor growth for a 1mm size due to the spin–spin relaxation of NV centers in FNDs [83].

Glioma is a special kind of brain tumor that originates in the glial cells surrounding and supporting the function of nerve cells. Generally, the treatment incorporates surgery, radiation therapy, and chemotherapy; however, the injected medicines cannot quickly proliferate the blood vessels surrounding the brain, resulting in the leakage of medication from the target area. FNDs as potential drug delivery agents, combined with chemotherapy medicine, doxorubicin (DOX), forming FND-DOX, have shown an enhanced uptake and retention of doxorubicin in glioma cells. FND-DOX was delivered effectively at the target using convection-enhanced diffusion [88]. Therefore, the effect of the medicine on the brain tumor cells can be significantly improved. The average cell viability for U-87 MG and U-251 MG treated with FNDs was above 90% after 72 h of treatment, exhibiting remarkable biocompatibility. The DOX uptake in U-251MG was doubled (1200 nmol/mg) after 4 h of treatment, and the DOX retention in U-251MG increased by 5 times (750 nmol/mg) after 24 h of treatment. The retention volume of ND-DOX was 1.1 cm3 after 72 h of treatment [88].

The biocompatibility and the possible effects on cell viability of surface-modified carboxylated nanodiamonds (cNDs) have been quantitatively determined by using human lung epithelial carcinoma, cancerous A549 cells, and fibroblasts cell line (HFL1) [89]. It is observed that the injection of (5, 10) nm size and a (0.1–100) µg/mL concentration of nanodiamonds quickly form an accumulation in the A549 cells without altering the cell viability and protein distribution. Under the same concentration, the cNDs were easily ingested in both types of cells, which was observed from the fluorescence emission. The fluorescence of 5 nm particles was perceived as higher than the 10 nm particles detected using confocal microscopy.

Human breast cancer (adenocarcinoma) is a malignant kind of cancer cell that is also considered a subtype of carcinoma. If the glandular cells start growing abnormally due to mutations in DNA replication, this could result in the formation of tumors that could further overcome the healthy tissues. Adenocarcinoma can be initially seen as a thick white membrane that can quickly spread and diffuse through the soft tissues. Adenocarcinoma is also dominant in other diseases, such as lung, breast, prostate, pancreatic, and colorectal cancers. The desired immobilization of a chemotherapy medicine such as DOX at the target tissue can be reasonably achieved by the ND-DOX composite acting as a cancer therapy platform [90]. The results show that the ND-carboxylic group combines with the DOX amino group forming stable non-covalent bonds at the FND surface. When the environment is acidic, the ND-carboxylic group ionized, and as a result, the DOX was released, successfully targeting the cancer cells. The study finds that ND-DOX is an effective candidate as a drug delivery material [90].

The imaging and detection of the in vivo growth of cancer is a primary tool for early stage detection and therapy. In this regard, magnetic resonance imaging (MRI) based on the measurement of RF-induced longitudinal and transverse spin-relaxation T_1_ and T_2_ weighted images can reliably observe the thickness of human tongue cancer [91]. Further, the contrast comparison of these spin-relaxation times can also be used for the diagnosis of rectal [92,93], prostate [94], and breast cancers [95,96]. The histological growth of tumors at different stages has been observed from 3 to 9 mm in size. The conjugation of magnetic nanoparticles with FNDs was used for contrast and resolution-enhanced nanoparticles-based magnetic resonance imaging [97,98,99]. FND-Mn^2+^ composite nanoparticles proved to be effective contrast enhancement agents in an MRI by implementing appropriate measurement sequences, indicating a cell viability approaching 100% for 0–100 µM concentrations for 24 h [100]. The DND-Mn composite also showed contrast enhanced by a factor of 7 after 120 min.

Gadolinium complex magnetic molecules combined with FNDs forming FND-Gd aggregates were used for in vivo tracking and the morphological analysis of cancer in mice for 26 days [101]. The results have shown a 300-times increase in cell delivery of Gd complexes at the target without altering the cellular toxicity. The observed cell viability exceeds 90% for the FND-Gd concentration range of 31–500 µg/mL [101]. The FND-Gd conjugation demonstrated high deliverability and a better MRI contrast than other carbon-based materials, such as graphene and carbon nanotubes.

The MRI uses the magnetization of randomly oriented protons in water inside the tissues. An intense uniform external magnetic field is applied to align the randomly oriented nuclear spins, subject to RF pulse excitation, altering the orientations of nuclear spins, which later relax back to their equilibrium position by emitting the absorbed RF energy. The acquired information in the form of images can be translated into grey shades displaying the intensity of an emitted signal depending upon the available protons’ density inside the tissue. The brightness and contrast of images can be accessed to identify the presence of tumors in the brain. The brightness and contrast of the acquired images can also be enhanced by injecting magnetic nanoparticles (Gadolinium) into the sample. T_1_ is evaluated as a time constant for which the excited nuclear spins regain an equilibrium state, whereas T_2_ determines the time constant for which the nuclear spins achieve dephasing in a direction perpendicular to the external magnetic field [102]. As a non-invasive technique, the MRI is preferred over X-ray imaging and a CT scan providing clear images of soft tissues [103]. The MRI can analyze various body parts, such as the chest, abdomen, pelvis, spinal cord, and brain [104,105,106,107]. In this context, the NV center in FNDs allows us to optically initialize, control, and detect the spin state similar to the operations on nuclear spins in the MRI. This notion has been widely exploited for optical magnetometry using a single and an ensemble of NV centers [27,108]. A diamond spin magnetic microscope enabling wide-field imaging of tumors with a spatial resolution of 1 µm in a 0.5 mm^2^ field of view has been reported using a bulk diamond crystal [109]. The target tissues labeled with 20 nm superparamagnetic nanoparticles (SMNPs) were deposited on the diamond surface for magnetic sensing with a spatial resolution of 400 nm. Under the influence of alternating magnetic fields, the SMNPs flip their magnetization, causing noticeable shifts in the NV-ODMR spectra within an NV-sensing volume of 0.1 µm^3^. The ODMR shift is then interpreted using a deep-learning algorithm to estimate the target protein distribution in immunomagnetic images [109]. The experiments were carried out using human lung tumors by labeling the proteins (EGFR, TfR, Ep-CAM, Ki67, and PD-L1) as cancer biomarkers exhibiting an SPMNs density of 300–600/µm^2^ in the magnetic images, which were further processed for cell segmentation and quantification. In the case of the PD-L1 protein, the maximum number of PD-L1 positive cells was 90%, deduced from the SMNPs density. Another study revealed the application of FNDs coated with nanogel as a nano heater upon irradiation with an NIR source (810 nm). This experiment reported a programmable cancer cell (HeLa) death by raising the intracellular temperature to 30 °C [67].

Alzheimer’s disease is a neurological disorder causing shrinkage in the size of the brain, leading to memory loss, confusion, poor judgment, depression, abrupt behavioral changes, and a decline in cognitive abilities. If untreated, it can also cause dementia. It is generally considered to happen due to an abnormal build-up of amyloid and tau proteins, obstructing the usual functions of brain cells by reducing the electrical signaling between the neurons. Unfortunately, there is no cure for this disease, and the annual cost of treatment is ($10,000–$35,000). Drugs such as Aducanumab (Aduhelm), Galantamine, Rivastigmine, and Donepezil are available to partially improve cognitive abilities for patients suffering from mild symptoms. In this situation, a difficult task in early stage detection is the in vivo diagnosis of neurological activity (cerebral metabolic rate) due to the unavailability of appropriate drugs that can penetrate the blood–brain barrier [110]. Functionalized FNDs attached with bifunctional peptides can penetrate and span the cellular membrane, identifying amyloid beta chunks as an active Alzheimer’s biomarker leading toward ultrasensitive detections [111]. This study also explored the presence of the 4G8 and Alexa-488 secondary antibodies in the hippocampus (mice brain) due to the strong luminesce of FNDs explored using confocal microscopy.

## 6. Application of FND Bioimaging with Artificial Intelligence

The aim of AI and machine-learning (ML) algorithms in medical science is to develop strategies for early stage disease diagnosis by analyzing clinical data using convolution neural networks and deep-understanding (DL) deterministic modeling. ML algorithms are already being tested for predicting symptoms of brain tumors, brain aging, and neural disorders.

ML algorithms have been empowering FNDs for intracellular magnetic microscopy [109]. Deep-learning algorithms enable the high-contrast reconstruction of magnetic images from optical images. The optical expression of biomarkers is successfully converted into a sufficient magnetic intensity. In this study, when labeled with magnetic nanoparticles, the sample tissues allowed the observation of the size, morphology, and growth of tumor cells. Fluorescence-based biosensors can significantly benefit from the potential uses of ML algorithms, such as rapid on-chip data processing, noise suppression, image analysis, and segmentation [112]. Integrating an AI-based processor with medical test strips can support the data acquisition and real-time monitoring for any non-linearity in the biosensor response under inevitable conditions or contamination.

Further extending the scope of AI-powered electronic chips, which can store and process various algorithms and access large online disease databases, can make rapid onsite testing using pattern recognition and alert the physicians for any possible anomalies during the clinical trials. Intracellular imaging using fluorescent biosensors faces challenges due to time-varying non-linear backgrounds. Artificial neural networks were used to solve the inverse problem of optical biosensing in chicken egg white [113]. After implementing the artificial neural networks, the optical signal of FNDs was successfully filtered out from the background autofluorescence under low concentrations of FNDs (2–3 µg/mL). With this approach, the accuracy of detection was enhanced by 1.5 times. A similar effort for reducing the background autofluorescence using egg protein was also reported [114]. The use of AI for FND-based drug development was discussed in [115]. The need for personalized drugs for diseases with diverse effects and lethal variants is pivotal. CURATE.AI is a clinically tested AI-based platform that assists medical experts in selecting drug and dose concentrations after analyzing the patient’s medical history [116]. The evaluation of AI for healthcare has been encouraged to achieve the desired outcome with minimal side effects.

**Figure 7:**
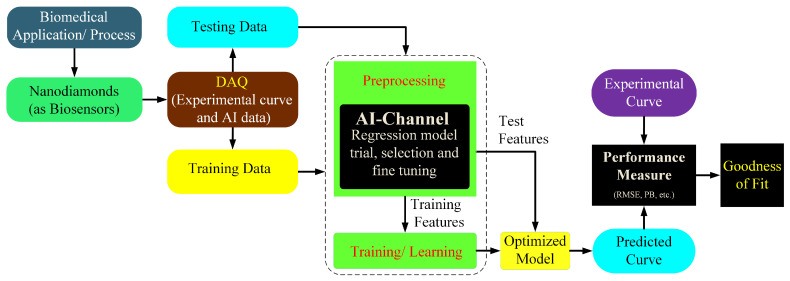
Illustration of AI scheme for the assessment and prediction of a biological process (BP). The proposed model relies upon the signal acquired from nanodiamonds which act as sensors providing electrical signals and an interface between BP and the data acquisition process. The accuracy of this model relies upon the comparison of the goodness of fit from the experimental and predicted curve based on root mean squared error values.

Currently, the main challenge is processing an extensive database for reliable results, demanding high computational resources and efficient algorithms providing accurate results. The fabrication of standard medicine is challenging and requires expert manpower, financial resources, and an unexpected time scale. In this regard, AI can significantly reduce the time and laborious effort by identifying the possible combinations of proteins and biomolecules, which could have better results and the least side effects. This idea can be further extended toward discovering appropriate biomarkers, which can then be utilized for disease diagnosis at different stages of progression and personalized drugs for individual cases. An example of this application where medical systems employing AI facilitated finding the optimized contents of a multi-drug conjugated with FNDs for curing human breast cancer was briefly examined in [117]. The efficiency and accuracy of this technique can be improved by using multiple data sources and sensors. An illustration of the model of the AI-based diagnosis is shown in Figure 7. Some of the AI’s tested and verified bioimaging applications associate brain imaging for detecting various types of tumors, such as glioma and Meningioma [118].

## 7. Future Challenges and Recommendations

Atomic defects in FNDs have now been established as an excellent platform for ultrasensitive optical biosensing under ambient conditions. So far, these biosensors have shown great potential for monitoring intracellular metabolism and the possibility of early stage disease detection, which in the case of cancer and HIV initiates contamination of the human body at the cellular level. The FND-Polydopamine composite emerges as a unique nano heater-thermometer that could be extended toward optically controlled targeted cancer therapy. However, further research for the improvement in purity and surface chemistry of FNDs is required to get the full benefit of its potential biosensing. A significant limitation in FND biosensing is the low optical collection efficiency and the need to keep the sensor near the target, which limits the sensitivity and speed of the measurement, reducing the volume of interest to be explored. A strong magnetic coupling between the NV centers and the neighboring paramagnetic impurities also degrades the acquired data due to magnetic noise. NV centers in small FNDs (<5 nm) suffer from optical instability and blinking. A vast majority of commercially available FNDs inside a selected batch display a lower fluorescence emission, due to which only the suitable candidates showing an acceptable signal-to-noise ratio are selected for biosensing. Similarly, the inversion process for the design of nanosensors from a predetermined state is based on the maximum sensitivity of the biomarkers being detected [119]. However, there are limitations to this inverse design process due to the constraints imposed on the composition, shape, and size of the nanomaterials.

## 8. Conclusions

This article discussed the recently reported biomedical imaging and sensing applications of FNDs. The advent of highly sensitive, efficient, and accurate techniques for infections and disease diagnosis in biological systems is a primary goal for understanding living microorganisms and developing effective medicines. Quantum sensors in FNDs deliver outstanding characteristics of intracellular thermometry and microscopy, which could lead us toward the possibility of understanding intracellular metabolic heat generation, intracellular signaling, and thermogenesis. The opportunity of FNDs surface modification and conjugation with numerous materials such as metallic nanoparticles, magnetic nanoparticles, proteins, antibodies, and various functional groups opens new routes for scientific research of understanding living organisms at the individual cell level. Hybrid nanoparticles can play an essential role in localized magnetic hyperthermia. The bulk diamond and nanocrystals implemented in the NV spin magnetic microscope have shown potential for sensing biomagnetism in magnetotactic bacteria and the electrical signaling in neurons. The nanoscale MRI has shown promising results by detecting single proteins attached to nuclear spins in diamonds. FNDs, when attached with magnetic Gadolinium complexes, have demonstrated the detection of SARS-CoV virus RNA (less than 100) in a one-second measurement time, achieving less than 1% of the false negative rate. The size-controlled FNDs have shown capabilities of enhancement in drug delivery in glioma cells and as a therapeutic agent for chemotherapy. The NV spin relaxometry has been utilized for the in vivo monitoring of tumor growth up to 1mm. Magnetically labeled biomolecules explored with NV spin relaxometry showed potential for detecting HIV-AIDS, cancer, and viruses attacking the respiratory system (Ebola and influenza). Despite the outstanding sensing capabilities, diamond nanosensing still faces challenges such as a low collection efficiency, magnetic noise from paramagnetic impurities, and high background autofluorescence inside biological species, reducing the achievable sensitivity. Surface impurities such as oxides and carbides could also induce undesired photothermal effects. Commercially available FNDs have also shown a wide size distribution and variation in brightness with a low quantum yield, suggesting that improvement in the fabrication of diamonds is required. New modalities for diamond biosensing are being developed, such as integrated devices, fiber coupling, and active manipulation via optical tweezers. Implementing ML algorithms has further strengthened NV-sensing and magnetic imaging by enabling superresolution microscopy with a spatial resolution of <50 nm.

## Figures and Tables

**Table 1:** Magnetometry with the highest achieved sensitivity using ensemble and single NVCs.

Sensing Type	Scheme	Sensitivity Achieved	References
Magnetic field	DC magnetometry Using ensembles of NV centers	164 pTHz^−1/2^	[47]
Magnetic field	CW-ODMR using ensembles of NV centers	1 nTHz^−1/2^	[48]
AC magnetic field at 20 KHz	Ensembles of NV centers	0.9 pTHz^−1/2^	[49]
AC magnetic field	Biological media (mouse brain)	100 pTHz^−1/2^	[50]

**Table 2:** A comparison between various fluorescent nanosensors [1].

Property	Organic-Dye	Quantum-Dot	NV Center
Size (nm)	~1	~10	~5
Emission band	UV-IR	UV-IR	600–800 nm
Absorption band (nm)	400–700	350–750	450–650
Observed quantum yield	~1	~0.8	~0.8
Photostability level	Low	High	Exceptionally High
Biocompatibility level	High	Low	High
Thermal sensitivity at room temperature (K^−1^)	0.02–0.3	10^−4^–10^−3^	0.01
Operating range (K)	287–465	100–400	290–400

## Data Availability

Not applicable.
